# Essentialist Biases in Reasoning About Emotions

**DOI:** 10.3389/fpsyg.2020.562666

**Published:** 2020-09-23

**Authors:** Iris Berent, Lisa Feldman Barrett, Melanie Platt

**Affiliations:** Department of Psychology, Northeastern University, Boston, MA, United States

**Keywords:** emotions, essentialism, innateness, embodiment, core cognition, naïve psychology

## Abstract

A large literature debates whether emotions are universal and innate. Here, we ask whether reasoning about such matters is shaped by intuitive Essentialist biases that link innateness to the material body. To gauge the perception of innateness, we asked laypeople to evaluate whether emotion categories will be recognized spontaneously by hunter–gatherers who have had no contact with Westerners. Experiment 1 shows that participants believe that emotions are innate and embodied (facially and internally) and these two properties correlate reliably. Experiment 2 demonstrates that the link is causal. When told that emotions are localized in specific brain areas (i.e., embodied), participants concluded that emotions are innate. Experiment 3 shows that this naïve view persists even when participants are explicitly informed that these emotions are acquired. Our results are the first to suggest that laypeople incorrectly believe that, if emotions are embodied, then they must be innate. We suggest that people’s failure to grasp the workings of their psyche arises from the human psyche itself.

## Introduction

A large literature debates whether emotions are universal and innate and, correspondingly, whether they are uniquely expressed in the face, body, and brain (e.g., Ekman et al., 1969; Tooby and Cosmides, 2008; Sauter et al., 2015; Siegel et al., 2018 vs. Gendron et al., 2018; Barrett et al., 2019, for proponents and opponents of innate emotions, respectively). Despite hundreds of studies on the topic, the debate shows no signs of abating. The factors that contribute to the persistence of the innateness debate are unknown. Here, we explore the possibility that the stalemate emanates from biases inherent in naïve psychology–the tacit beliefs that people hold about the bodies and the minds of others (Baron-Cohen et al., 1985; Keil, 1986; Spelke, 1994). Very little research has specifically addressed the role of naïve psychology in views of emotion. To begin investigating this question, we examined laypeople’s beliefs about whether emotions are universally expressed in the material body and whether such beliefs are linked to their reasoning about innateness.

Previous research has shown that adults rate emotions highly on biological attributes such as “naturalness,” akin to bodily state and natural kind categories, as distinct from abstract nominal kinds and cognitive states (Lindquist et al., 2013). These observations suggest that laypeople’s understanding of emotions is guided by Essentialism–an intuitive psychological principle that governs reasoning about living things (Keil, 1986; Gelman, 2003). Per Essentialism, living things are believed to possess an innate immutable essence that transfers from parent to their offspring (Gelman, 2003). For example, young children know that racoons cannot turn into skunks by painting their exteriors, as such manipulations do not alter their inherent essence (Keil, 1986). Children also know that a baby rabbit raised by monkeys will maintain the properties of its biological kind (e.g., it will grow up to exhibit long ears and eat carrots, rather than have short ears and eat bananas; Gelman and Wellman, 1991). These observations suggest that, in naïve biology, innate biological traits must possess a special immutable essence.

In addition, a careful read of the literature on essentialism suggests that people further believe this innate essence must form part of the material body (for review, Berent, 2020). Children, for example, assert that a puppy inherits its brown color from its mother by the transfer of some tiny brown pieces of matter (Springer and Keil, 1991). Children further believe that this innate essence is physically localized in the material body (i.e., the body of the puppy and its mother). Furthermore, the essence is presumed to be in a specific place within the body. So when children are invited to discover which animal is hidden inside a fossil, they insist that the sample be taken from the center of the fossil, but they do not do the same when interrogating non-organic material, such as rocks or metals (Newman and Keil, 2008). Other findings suggest that material bodily traits (e.g., foot size) are more readily perceived as innate than psychological characteristics (e.g., shyness, Heyman and Gelman, 2000). These results suggest that, in naïve biology, not only are innate traits embodied, but also conversely, embodied biological traits are innate.

This Essentialist belief provides a powerful causal mechanism for reasoning about the innateness of psychological traits, such as emotions. If people assume that (a) innate traits must possess an inborn essence and (b) the essence must form part of the material body (i.e., essences are *embodied*), then their tendency to interpret psychological traits as innate will depend on their perceived materiality and embodiment. Traits that are perceived as immaterial and disembodied should be viewed as ones that are unlikely to be innate (i.e., a negative innateness bias), whereas those that are readily linked to the material body would be presumed innate (i.e., a positive innateness bias).

The contrast between knowledge (epistemic states, such as knowing that “objects are cohesive”) and emotions (e.g., “anger”) allows us to evaluate this prediction. Intuitively, emotions appear to be embodied in the material body (e.g., in the face), whereas knowledge seems disembodied and immaterial. If our intuitive nativist intuitions are guided by our beliefs about the materiality of psychological traits, then we expect people to exhibit opposite nativist intuitions toward knowledge and emotions (Berent et al., 2019a; Berent, 2020).

Recent studies from our lab have evaluated people’s nativist intuitions toward knowledge. We found that people presume that knowledge is not innate, and they maintain this bias despite explicit evidence to the contrary (Berent et al., 2019b; for converging evidence, see Wang and Feigenson, 2019). Moreover, the perception of knowledge as innate was linked to its perceived materiality. Specifically, epistemic states tended to be viewed as immaterial–the less material the trait, the less likely it was to be viewed as innate (Berent et al., in press). Thus, for epistemic traits (i.e., knowledge), people are negatively biased against innateness (Berent et al., 2019b, in press).

Here we test for the complementary (positive) bias for emotions. Observing that a given emotion category (e.g., fear) is expressed in the body (e.g., in the face), people would presume that this emotion is innate. Moreover, if in intuitive psychology, most emotions are embodied, then people would tend to view emotions as innate. We hypothesize that this embodiment–innateness link is causal: people are inclined to view emotions as innate precisely *because* they believe that emotions are embodied.

Our three experiments test this hypothesis. Experiment 1 asked whether the (perceived) innateness of an emotion category (e.g., fear) is linked to its propensity to be expressed in the human face and body. Experiment 2 determined whether this association between embodiment and innateness is causal, such that people are more likely to believe that emotions are innate when these emotions are described as embodied (i.e., localized in specific brain regions) compared to when they are not (i.e., devoid of brain localization). We predicted that embodiment would promote the perception of emotions as innate. Experiment 3 investigated whether people maintain their conviction that emotions are innate even when informed that the emotions in question are acquired.

Following Samuels (Samuels, 2004), we define psychological innateness as the propensity for spontaneous emergence, without reliance on learning. To gauge innateness, our experiments thus invited participants to determine whether a given emotion will be recognized spontaneously by a hunter–gatherer who has had no interaction with Western people (i.e., no opportunity for learning). We reasoned that, if the emotion is considered innate, then it should be perceived as one that is likely to emerge spontaneously in the hunter–gatherer, hence, as potentially available to all humans (i.e., universal).

We chose this approach over explicit innateness ratings for two reasons. First, we believe this tacit measure is more likely to tap into laypeople’s tacit nativist intuitions. Second, this very same approach has been amply used in past research in affective science (Ekman et al., 1969; Sauter et al., 2010; Gendron et al., 2014). While our present research decidedly concerns laypeople’s intuitions, rather than scientists’, a convergence would open up the possibility that the relevant biases might apply more generally. Our present investigation seeks to determine whether laypeople’s nativist intuitions concerning emotions depend on their perceived embodiment.

## Experiment 1: Emotions Are Viewed as Embodied and Innate

Participants in Experiment 1 rated how likely emotions are to (a) be expressed in the face, (b) elicit a physiological response in the body, and (c) be recognized spontaneously by hunter–gatherers who have had no previous contact with Westerners (suggesting that emotions are innate and universal). We hypothesized that people would consider emotions as embodied and innate and that beliefs about the innateness of emotions would be associated with beliefs about their embodiment.

### Methods

#### Participants

Sixty participants took part in this experiment. In this and all subsequent experiments, participants were recruited from Amazon Mechanical Turk. They were all adult (18 years or older) native English speakers who resided in the United States. All participants were reportedly free of reading disorders and had reportedly taken no upper level courses (beyond an introductory level) in Psychology. In experiment 1, 37% of the sample had a high school education, 50% had a college education, and 13% had a postgraduate education.

To be included in the sample, participants in Experiment 1 must have further provided a coherent answer to a question presented at the end of the experiment (asking them to explain their response) and spent at least 200 s on the experiment. All participants signed a written informed consent form, approved by the Institutional Review Board of Northeastern University.

Sample sizes in Experiments 1–3 were determined by pilot work as well as by previous research (e.g., Lindquist et al., 2013). Accordingly, we determined that samples of at least *N* = 60 per group were optimal to yield modest to large effect sizes.

#### Materials and Procedures

The materials consisted of twenty emotion terms (*anger*, *disgust, fear, happiness, surprise, love, contentment, excitement, joy, pride, sadness, shame, contempt, jealousy, relief, desire, embarrassment, pain, awe*, and *boredom*). This word list was compiled from Lindquist et al. (2013) and Cordaro et al. (2018). The 20 emotion terms were presented in random order. For each emotion term, participants were asked to consider three questions (counterbalanced for order, see Supplementary Appendix 1).

One question asked participants “how likely is it that a person who feels this emotion will “show” it as a distinct facial expression? In other words, how likely is it that you could tell that a person is experiencing this particular emotion from the person’s facial expression?” A second question asked, “how likely is it that each emotion will elicit a physical bodily response (e.g., a change in blood pressure, heart rate, perspiration)?” To evaluate the universality of emotions (a gauge of innateness), a third question invited participants to consider an experiment involving people in a small-scale society living in a remote part of the world, following the methods of actual published experiments (e.g., Ekman et al., 1969; Ekman and Friesen, 1971). Participants were told that “these people are hunter-gatherers; they have no electricity, so they have no access to media, and most of them have had no interactions with Westerner people before.” They were then asked to reason “how likely is it that they would recognize an emotion from the list below in facial expression. For concreteness, suppose you presented them (with the help of an interpreter) with a short story, depicting an event (e.g., “a person encounters a threatening animal in the jungle and he is afraid for his life”). Next, you showed them two pictures, depicting two distinct facial expressions (“horror” vs. “euphoria”), and asked them to pick which picture corresponds to the person depicted in the previous story. How likely is it that their responses would match those of United States participants?” For each of the three questions, participants provided a rating for each of the 20 emotion words on a seven-point scale (1 = very unlikely; 4 = neither likely nor unlikely; 7 = very likely).

### Results

**FIGURE 1:**
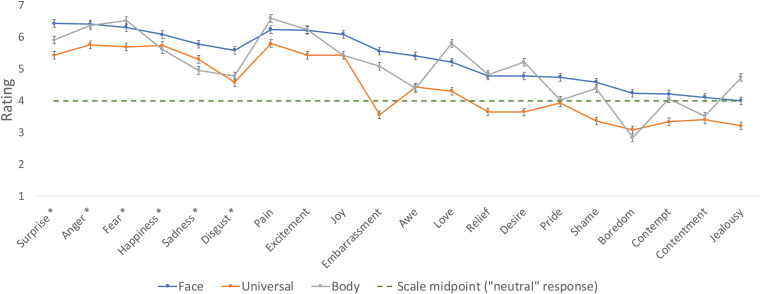
The mean rating of emotions with respect to their manifestation in the face and body, and their universal recognition. “Basic” emotions are marked by asterisks. Error bars are 95% CI for the difference between the means. The “neutral” midpoint of the rating scale (4) is indicated by the dashed line.

Figure 1 plots participants’ rating of how likely each emotion is to be expressed in the face, manifest in the body, and emerge universally.

We first compared the mean response to each of these three questions against the scale’s neutral midpoint (4-“neither likely/unlikely”) using a paired *t*-test. To evaluate the generality of our conclusions across participants and items (specific emotions), we conducted these tests using both participants (*t*_1_) and items (*t*_2_) as random effects.

The results confirmed that people considered these emotions as likely to imprint their marks on both the face [*M* = 5.33, *t*_1_(59) = 13.00, *p* < 0.0001, d = 1.68; *t*_2_(19) = 7.04, *p* < 0.0001, *d* = 1.57] and the body [*M* = 5.06, *t*_1_(59) = 9.91, *p* < 0.0001, *d* = 1.28; *t*_2_(19) = 4.64, *p* < 0.0002, *d* = 1.04]. Similarly, people believed these emotions would be likely recognized spontaneously by hunter–gatherers, a result significant by participants [*M* = 4.45, *t*_1_(59) = 3.48, *p* < 0.001, *d* = 0.45] and marginally so by items [*t*_2_(19) = 1.97, *p* < 0.07, *d* = 0.44]. This finding suggests that participants tended to view these emotions as innate and universal.

Strikingly, the presumed innateness of these emotions (gauged by their universality) correlated with beliefs about their propensity to manifest in the face [*r*_1_(58) = 0.44, *p* < 0.0005; *r*_2_(18) = 0.94, *p* < 0.0001] and in the body [*r*_1_(58) = 0.39, *p* < 0.003; *r*_2_(18) = 0.81, *p* < 0.0001]. Additionally, beliefs about the face and body manifestations correlated strongly [*r*_1_(58) = 0.71, *p* < 0.0001; *r*_2_(18) = 0.83, *p* < 0.0001].

To compare laypeople’s intuition and distinctions made in affective science, we further examined people’s rating for certain emotion categories that are classically proposed as universal “basic emotions” (Anger, Disgust, Fear, Happiness, Sadness, and Surprise; classified following Ekman, 1992; Sauter et al., 2010) and “non-basic emotions.”

The 2 Emotion (Basic/Non-basic) × 3 Question (face/body/innateness) ANOVA yielded a significant effect of Question [*F*_1_(2,118) = 25.81, *p* < 0.0001, η^2^ = 0.09; *F*_2_(2,36) = 21.49, *p* < 0.0001, η^2^ = 0.10], as the universality question elicited a lower rating than both the facial and bodily questions (Tukey HSD, *p* < 0.0001; *p* < 0.0001, respectively, by both participants and items).

Critically, the main effect of Emotion type was highly significant [*F*_1_(1,59) = 200.95, *p* < 0.0001, η^2^ = 0.23; *F*_2_(1,18) = 8.84, *p* < 0.009, η^2^ = 0.27], as putatively basic emotions (*M* = 5.72) were rated higher than non-basic emotions (*M* = 4.61). The interaction between Emotion and Question was marginally significant [*F*_1_(2,118) = 6.41, *p* < 0.003, η^2^ = 0.01; *F*_2_(2,36) = 1.82, *p* < 0.18, η^2^ = 0.01], as the effect of Emotion type was largest for the innateness question Δ = 1.37 (for the face and body questions, Δ = 1.08 and Δ = 0.89, respectively). Thus, laypeople’s beliefs in the embodiment of emotions and their innateness were particularly pronounced for putatively basic emotions.

Summarizing, Experiment 1 shows that, overall, people tended to view emotions as both innate and embodied, especially for categories that are presumed to be biologically “basic.” The perception of innateness, moreover, correlated with the belief that emotions are expressed in both the face and the body. We hypothesized that when people view emotions as embodied, they also perceive them as innate *precisely because* they interpret embodiment as evidence for innateness. Experiment 2 tests this possibility.

## Experiment 2: Embodiment Promotes the Perception of Emotions as Innate

To determine whether the perception of emotions as innate is caused by the belief that they are expressed in the material body, Experiment 2 manipulated beliefs about the embodiment of emotions. Embodiment, here, was operationalized by the instantiation of an emotion in a particular brain area, corresponding to a discrete piece of matter. Of interest is whether describing an emotion as embodied will promote its view as innate.

Embodiment was manipulated across two groups of participants. Both groups were presented with the same list of 20 emotion words from Experiment 1. The “material” group was informed that these emotions each are localized in a distinct region of the brain. Participants in the “immaterial” condition were informed that these emotions are devoid of a brain localization. If people believe that embodied emotions are innate, then the “innateness” rating should be higher in the “material” relative to the “immaterial” condition.

### Methods

#### Participants

Two groups (*N* = 60 each) took part in the experiment. To be included in the sample, participants must have provided a coherent answer to a question presented at the end of the experiment (asking them to explain their response) and spent at least 150 s on the experiment (the time cutoff was decreased because the duration of Experiment 2 was shorter than Experiment 1). Four participants (three in the “material” condition) were removed from the analyses because their mean responses fell over 2SD from the group mean. In one group (the “material” group), 35% of the sample had a high school education, 52% had a college education, and 13% had a postgraduate education. In a second (“immaterial”) group, 40% of the sample had a high school education, 37% had a college education, and 23% had a postgraduate education. A comparison of the education levels in the two groups (operationalized on a 1–3 scale; 1 = high school, 2 = undergraduate training; 3 = graduate training) yielded no significant difference (*t* < 1).

#### Methods and Procedure

As in Experiment 1, participants were asked to assist a scientist in determining which emotions are likely to be recognized from their facial expressions by a hunter–gatherer who has had no previous contact with Westerners. These emotions were the same 20 emotions in Experiment 1 (putatively basic vs. non-basic, as defined therein). Prior to reading this question, however, participants were informed about the localization of these emotions in the brain (see Supplementary Appendix 1).

Participants in the “***material***” condition were told that past research has shown that “when people engaged in that emotion, that region of the brain was active, and different emotions activated different regions. The scientist concluded that each of these emotions is associated with a specific material localization in the human brain”. In the “***immaterial***” condition, participants were told that, in previous research “the scientist was not able to link any of these emotions with a specific brain region. So, when people engaged in each of these emotions, no distinct region of the human brain was activated, and the pattern of activation for different emotions was overlapping. Accordingly, the scientist concluded that these emotions are not associated with any specific localization in the human brain; in fact, the scientist is wondering whether these emotions even have a material basis in the human body”.

With this information in mind, participants in both groups were asked to determine how likely it is that a hunter–gatherer who has had no previous interactions with Westerners would be able to match facial expressions to a sentence. For example, the hunter–gatherers would be presented with the sentence “*a person encounters a threatening animal in the jungle and he is afraid for his life*” and asked to indicate which of two facial emotions (“horror” vs. “euphoria”) matches the person depicted in the story. Participants were once again reminded that these two emotions either do/do not correspond to distinct brain regions, as do the remainder of the emotions on the list. They were asked to rate how likely is it that the responses of the hunter–gatherers would match those of United States participants, for each of the 20 emotions (1 = very unlikely; 7 = very likely).

### Results

**FIGURE 2:**
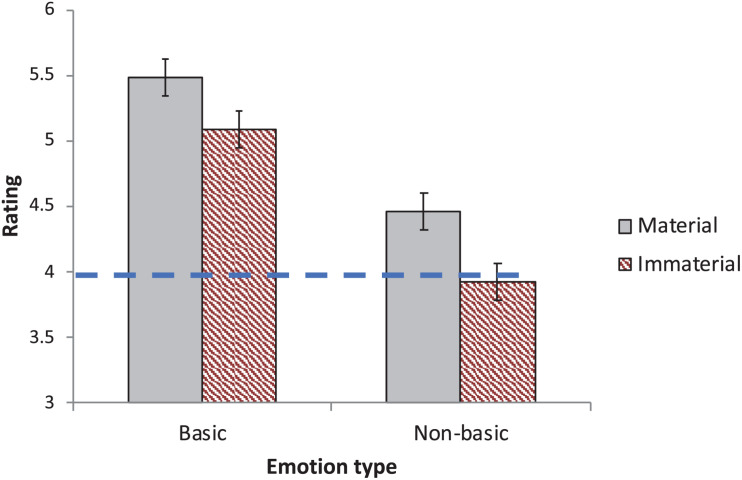
The effect of materiality on the perception of emotions as innate (in Experiment 2). Innateness is gauged by the spontaneous emergence of emotions in hunter–gatherers. The scale’s midpoint is indicated by the dashed line. Error bars are 95% CI for the difference between the means.

Figure 2 plots the effect of materiality on perception of emotions as innate (as gauged by their presumed universality). An inspection of the means suggests that participants rated emotions as more likely to be innate in the “material”–relative to the “immaterial” condition.

A 2 Materiality × 2 Emotion (Basic/Non-basic) ANOVA yielded a reliable main effect of Emotion type [*F*_1_(1,114) = 114.19, *p* < 0.0001, η^2^ = 0.16; *F*_2_(1,18) = 13.02, *p* < 0.003, η^2^ = 0.38], as putatively basic emotions were rated higher than non-basic emotions. Crucially, the effect of Materiality was significant [*F*_1_(1,114) = 5.38, *p* < 0.03, η^2^ = 0.03; *F*_2_(1,18) = 49.51, *p* < 0.0001, η^2^ = 0.07], and it did not further interact with Emotion type [*F*_1_(1,114) = 0.51, *p* < 0.48, η^2^ = 0.00; *F*_2_(1,18) = 1.19, *p* < 0.29, η^2^ = 0.00]. Thus, the presentation of an emotion as material promotes its perception as innate.

A single-sample *t*-test found that the mean rating of all 20 emotions was significantly higher than the scale’s “neutral” midpoint for the “material” condition [*M* = 4.77, *t*_1_(56) = 5.37, *p* < 0.0001, *d* = 0.71; *t*_2_(19) = 4.44, *p* < 0.0003, *d* = 0.99]. In the “immaterial” condition, this difference did not reach significance [*M* = 4.27, *t*_1_(58) = 1.80, *p* < 0.08, *d* = 0.23; *t*_2_(19) = 1.47, *p* < 0.16, *d* = 0.33].

Thus, when told that emotions are localized in the brain, participants were more likely to conclude that these emotions are innate compared to when the same emotions were presented as devoid of material manifestation in the body. Experiment 3 further evaluates the strength of this conviction.

## Experiment 3: Our Nativist Bias

Experiment 3 examined whether the belief in innate emotions is a bias. We were especially interested in the degree of bias present for putatively basic emotions–the emotions that people are prone to view as embodied and innate. Will people maintain their conviction that these emotions are likely innate even when *explicitly* informed that these emotions are actually acquired?

### Methods

#### Participants

Sixty participants took part in this experiment. To be included in the sample, participants must have provided a coherent answer to a question presented at the end of the experiment (asking them to explain their response) and spent at least 150 s on the experiment. In Experiment 3, 22% of the sample had a high school education, 62% had a college education, and 17% had a postgraduate education.

#### Materials

Participants were asked to reason about the propensity of a group of isolated hunter–gatherers to spontaneously recognize 20 emotions (putatively basic and non-basic) using the same emotion words and procedure as in Experiment 1. These participants, however, were further told that “previous research has conducted the same experiment in various cultures, and the results in different groups did not turn out the same. Accordingly, the scientist believes that people learn these emotions from experience with members of their own culture.” With this information in mind, participants were asked to determine “how likely is it that, when presented with each emotion below, the responses of the indigenous group would match those of participants in the United States?”. Full instructions are provided in Supplementary Appendix 1.

### Results

**FIGURE 3:**
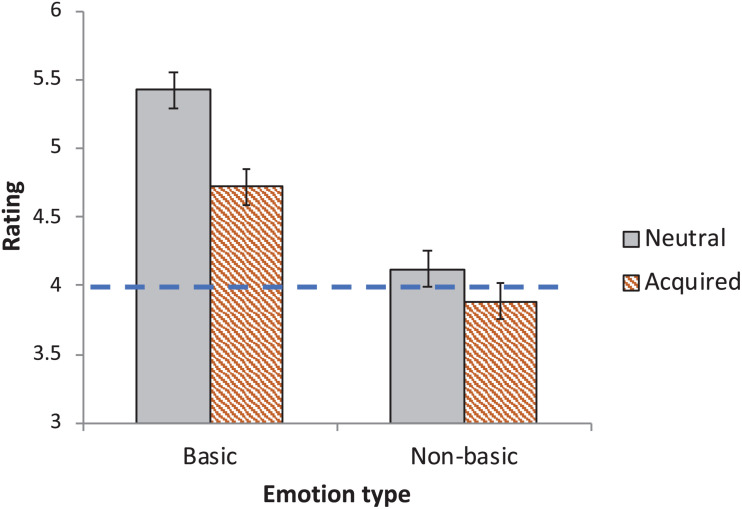
The perceived innateness of emotions as a function of their origins (Acquired vs. Neutral, in Experiment 3). Innateness is gauged by the spontaneous emergence of emotions in hunter–gatherers. The scale’s midpoint is indicated by the dashed line. Error bars are 95% CI for the difference between the means.

When informed that emotions are “acquired,” the mean rating of “non-basic” emotions (*M* = 3.85) no longer differed reliably from the scale’s midpoint [*t*_1_(59) = −0.98, *p* > 0.33, *d* = 0.13; *t*_2_ (13) = −0.93, *p* > 0.37, *d* = 0.25]. For putatively basic emotions, however, the mean “innateness” rating (*M* = 4.67) was still higher than the scale’s “neutral” midpoint, and this difference was highly reliable [*t*_1_(59) = 3.75, *p* < 0.0005, *d* = 0.48; *t*_2_(5) = 2.94, *p* < 0.04, *d* = 1.20, see Figure 3].

To confirm that participants did not simply ignore the instructions, we further compared their responses to participants in Experiment 1 (*N* = 60), who were given no information about innateness (a “Neutral” condition)^1^. We were specifically interested in responses for the putatively “basic” emotions. Since people do not necessarily view “non-basic” emotions as innate, the effect of the “acquired” instructions here might be minimal. Putatively basic emotions, on the other hand, are typically viewed as innate. If people have attended to the instructions, then participants in the “acquired” condition should view these emotions as less likely to be innate relative to the “neutral” condition. Figure 3 plots the results.

The 2 Group (Acquired/Neutral) × 2 Emotion (Basic/Non-basic) ANOVA yielded a reliable main effect of Emotion [*F*_1_(1,118) = 153.54, *p* < 0.0001, η^2^ = 0.17; *F*_2_(1,18) = 10.97, *p* < 0.004, η^2^ = 0.33], as well as a main effect of Group [*F*_1_(1,118) = 5.24, *p* < 0.03, η^2^ = 0.03; *F*_2_(1,18) = 20.89, *p* < 0.0003, η^2^ = 0.06]. The Emotion × Group interaction was significant [*F*_1_(1,118) = 9.72, *p* < 0.003, η^2^ = 0.01; *F*_2_(1,18) = 7.38, *p* < 0.02, η^2^ = 0.02].

Planned contrasts indicated that participants rated the putatively “basic” emotions significantly lower in the “acquired” relative to the “neutral” conditions [*t*_1_(161.33) = 3.34, *p* < 0.002, *d* = 0.578; *t*_2_(21.36) = 4.22, *p* < 0.0004, *d* = 1.47]; rating for “non-basic” emotions did not differ [*t*_1_(161.33) < 1; *t*_2_(21.36) = 1.68, *p* < 0.11].

The lower rating for the “basic” emotions in the “acquired” condition confirms that participants did heed the instructions. However, participants nonetheless maintained that emotions (especially, “basic” emotions) are likely to be recognized spontaneously by hunter–gatherers and Westerners alike. Thus, despite explicit instructions to the contrary, people still viewed these emotions as innate.

## General Discussion

Our investigation examined laypeople’s beliefs regarding the innateness of emotions. To gauge innateness, we asked people to determine whether emotions would be spontaneously recognized by a group of hunter–gatherers who have had no experience with Westerners. We hypothesized that people will be more likely to view emotions as innate when they believe these emotions are manifested in the material body.

In line with this hypothesis, Experiment 1 showed that people tend to view emotions as both embodied and innate, and these two traits were reliably correlated. Experiment 2 demonstrated that the links between embodiment and innateness are causal. We found that when people are informed that emotions are embodied (i.e., localized in the brain), they rate emotions higher for innateness (relative to participants informed that the same emotions are devoid of a material embodiment in the brain). Finally, in Experiment 3, we showed that people maintain their conviction that “basic” emotions are more likely to be innate even when told that these emotions were actually acquired. Together, these results suggest that the belief in innate emotions is a bias, and it is causally linked to the view of emotions as embodied.

We note that the bias in question is modest and relative: people did not categorically state that embodied emotions are unquestionably innate, nor were they absolutely confident that acquired basic emotions are inborn. Nonetheless, participants were systematically *more likely* to view emotions as innate when these emotions were presented as embodied (in Experiment 2), and, when informed that emotions are acquired, they still tended to view basic emotions as inborn (i.e., innateness ratings were above “neutral,” in Experiment 4). As such, our findings suggest that participants’ beliefs regarding the origins of emotions were biased: they presumed that embodied emotions are likely inborn.

We attribute this bias to intuitive Essentialism. Past research has shown that children believe that living things possess innate immutable essence, and this essence is embodied in the material body (Springer and Keil, 1991; Heyman and Gelman, 2000; Newman and Keil, 2008). Other evidence for the view of people’s essence as material is presented by their capacity to elicit contagion by physical contact (Rozin and Nemeroff, 2002). If people assume that innate natural traits (biological and psychological) must be embodied, then they would be more likely to interpret traits that they consider embodied as innate. Since people readily link “basic” emotions with bodily changes, they might be inclined to presume that emotions are innate. The results of Experiments 1–3 are in line with this possibility.

The positive bias we have uncovered toward innate emotions complements our previous findings, where we found a negative bias toward innate knowledge. As noted (in the section “Introduction”), people tend to view knowledge as immaterial and disembodied, a perception that contrasts with their view of emotions (as material and embodied). If Essentialism requires innate traits to be material, then we expect people to assume that (immaterial) knowledge cannot be innate. This prediction was borne out by our findings (Berent et al., in press, 2019b). Thus, the principle we have outlined to explain our positive nativist intuitions toward emotions can also account for our negative nativist attitudes toward knowledge.

Returning to the case of emotions, we note that our findings from Western participants cannot speak to the generality of this putative bias across cultures. Additionally, these results strictly concern naïve psychology–they do not evaluate whether emotions are in fact embodied or innate. These limitations notwithstanding, our results show for the first time that participants tend to systematically conflate emotions with their bodily manifestations (e.g., they believe that happiness is equivalent to a “Duchenne” smile), and to misinterpret those bodily changes as evidence for innateness.

To reiterate, we do not assert that people are necessarily mistaken in their *conclusion* that basic emotions are innate–as noted, this question remains a matter of ongoing controversy in affective science. Rather, we assert that laypeople rely on a mistaken *logic*. They incorrectly presume that the innateness of an emotion category can be discerned from its embodiment. Thus, if an emotion (e.g., fear) is perceived to “show up” in the face or correspond to a specific brain region, then the emotion category in question is innate. Given that embodied brain states can demonstrably arise either innately or by learning, the logic that “if it’s the body (e.g., brain) it’s inborn” is simply wrong.

Our findings suggest that laypeople systematically fail to grasp the workings of their own psyche. Ironically, these errors may well arise from principles that lie deep within the human psyche itself (Berent, 2020). These conclusions shed new light on human nature. Our results from laypeople do not speak to the question of whether similar biases might plague scientists. However, inasmuch as scientists are human, the discovery of such biases in human cognition suggests caution in the discussion of these matters within affective science.

## Data Availability Statement

Data is available on Open Science Framework: https://osf.io/e9bms/?view_only=cd3bf95f345b4fbdbd3049546877a84e.

## Ethics Statement

The studies involving human participants were reviewed and approved by the Institutional Review Board of Northeastern University. The patients/participants provided their written informed consent to participate in this study.

## Author Contributions

IB conceived the study, designed the materials, and wrote the initial draft. LF commented on the draft and the analyses. MP implemented the study, analyzed the data, and commented on the draft. All authors contributed to the article and approved the submitted version.

## Conflict of Interest

The authors declare that the research was conducted in the absence of any commercial or financial relationships that could be construed as a potential conflict of interest.

## Footnotes

1 Participants in Experiment 1 and Experiment 3 did not differ reliably on their overall level of education [*t*(59) = 1.58, *p* < 0.12].
