# Supplementary material for: Essentialist Biases in Reasoning About Emotions
Source: Front Psychol. 2020 Sep 23;11:562666. doi: 10.3389/fpsyg.2020.562666 (PMC7538619; doi:10.3389/fpsyg.2020.562666)
Supplement: Supplementary file 1 [file Data_Sheet_1.docx]

**Essentialist biases in reasoning about emotions**

**Supplementary Materials**

Iris Berent*^1^*

[i.berent@neu.edu](mailto:i.berent@neu.edu)

Lisa Feldman Barrett*^1^*

[L.Barrett@northeastern.edu](mailto:L.Barrett@northeastern.edu)

Melanie Platt*^1^*

[me.platt@northeastern.edu](mailto:me.platt@northeastern.edu)

^1^Department of Psychology

Northeastern University

Declarations of interest: none

Key words: Emotions, Essentialism, Innateness, Embodiment; Core cognition; Naïve psychology.

**Address for correspondence**

Iris Berent

Department of Psychology

Northeastern University

125 Nightingale Hall

360 Huntington Ave.

Boston MA 02115

[i.berent@neu.edu](mailto:i.berent@neu.edu)

Phone: (617) 373 4033

Fax: (617) 373-8714

**Appendix 1:**

**Instructions**

**Experiment 1**

**Emotions are viewed as embodied and innate**

In this experiment, we are going to present you with 20 emotion words, and ask you to judge each word with respect to three different questions.

1. How likely is it that a person who feels this emotion will “show” it as a distinct facial expression? In other words, how likely is it that you could tell that a person is experiencing this particular emotion from the person’s facial expression? Please address this question for each of the 20 emotions on the list.
2. Suppose you studied an indigenous group in a remote part of the world. These people are hunter-gatherers; they have no electricity, so they have no access to media, and most of them have had no interactions with Westerner people before. We ask you to reason how likely is it that they would recognize an emotion from the list below in facial expression. For concreteness, suppose you presented them (with the help of an interpreter) with a short story, depicting an event (e.g., “a person encounters a threatening animal in the jungle and he is afraid for his life”). Next, you showed them two pictures, depicting two distinct facial expressions (“horror” vs. “euphoria”), and asked them to pick which picture corresponds to the person depicted in the previous story. ***How likely is it that their responses would match those of U.S. participants?***

Please address this question for each of the 20 emotions on the list.

1. How likely is it that each of these emotions will elicit a physical bodily response (e.g., a change in blood pressure, heart rate, perspiration)? Please address this question for each of the 20 emotions on the list.

**Experiment 2**

**Embodied emotions are viewed as innate**

In this experiment, we are asking you to advise a scientist who wishes to determine the origins of various human emotions. The scientist wishes to examine the origin of ***two*** sets of human emotions. We ask you to advise the scientist in his investigation. Thank you!

****Note this is a between design, but participants are told that there are two sections.*

**Emotions that are localized in the brain (Condition A)**

Here is one list of human emotions considered by the scientist. In previous research, the scientist has examined how the brain gives rise to these emotions. The scientist was able to link each of these emotions with a specific brain region. When people engaged in that emotion, that region of the brain was active, and different emotions activated different regions. The scientist concluded that each of these emotions is associated with a specific material localization in the human brain.

The scientist is now interested to find out how likely it is that a person would spontaneously recognize this emotion in a facial expression. Suppose the scientist studied an indigenous group in a remote part of the world. These people are hunter-gatherers; they have no electricity, so they have no access to media, and most of them have had no interactions with Westerner people before.

For concreteness, suppose the scientist presented them (with the help of an interpreter) with a short story, depicting an event (e.g., “a person encounters a threatening animal in the jungle and he is afraid for his life”). Next, he showed them two pictures, depicting two distinct facial expressions (“horror” vs. “euphoria”), and asked them to pick which picture corresponds to the person depicted in the previous story. We now ask you to help the scientist predict how the hunter-gatherers will perform on this task for the 20 emotions listed below. Remember that the scientist believes that *each of the emotions on the list below would activate a specific brain region, distinct from those activated for other emotions.*

With this information in mind, consider again the emotion recognition task (e.g., which of two facial expressions depicts “horror”?). **How likely is it that the responses of the hunter-gatherers would match those of U.S. participants, for each of the 20 emotions below?**

Please indicate your answer on a 1-7 scale (1= very unlikely; 7= very likely).

Thank you!

**Emotions that are not localized in the brain (Condition B)**
Here is one list of human emotions considered by the scientist. In previous research, the scientist has examined how the brain gives rise to these emotions. The scientist was not able to link any of these emotions with a specific brain region. So, when people engaged in each of these emotions, no distinct region of the human brain was activated, and the pattern of activation for different emotions was overlapping. Accordingly, the scientist concluded that these emotions are not associated with any specific localization in the human brain; in fact, the scientist is wondering whether these emotions even have a material basis in the human body.

The scientist is now interested to find out how likely it is that a person would spontaneously recognize this emotion in a facial expression. Suppose the scientist studied an indigenous group in a remote part of the world. These people are hunter-gatherers; they have no electricity, so they have no access to media, and most of them have had no interactions with Westerner people before.

For concreteness, suppose the scientist presented them (with the help of an interpreter) with a short story, depicting an event (e.g., “a person encounters a threatening animal in the jungle and he is afraid for his life”). Next, he showed them two pictures, depicting two distinct facial expressions (“horror” vs. “euphoria”), and asked them to pick which picture corresponds to the person depicted in the previous story. We now ask you to help the scientist predict how the hunter-gatherers will perform on this task for the 20 emotions listed below. Remember that the scientist *was not able to link any of the emotions on the list below to a specific brain region, distinct from those activated for other emotions.*

With this information in mind, consider again the emotion recognition task (e.g., which of two facial expressions depicts “horror”?). **How likely is it that the responses of the hunter-gatherers would match those of U.S. participants, for each of the 20 emotions below?**

Please indicate your answer on a 1-7 scale (1= very unlikely; 7= very likely).

Thank you!

**Experiment 3**

**Our nativist bias**

In this experiment, we are asking you to advise a scientist who wishes to determine the origins of various human emotions. We ask you to advise the scientist in his investigation.

The scientist is planning to study emotions in two groups. One group consists of participants in the US. Another group consists of indigenous people in a remote part of the world. These people are hunter-gatherers; they have no electricity, so they have no access to media, and most of them have had no interactions with Westerner people before.

For concreteness, suppose the researcher presented participants (with the help of an interpreter) with a short story, depicting an event (e.g., “a person encounters a threatening animal in the jungle and he is afraid for his life”). Next, participants would be shown two pictures, depicting two distinct facial expressions (e.g., “horror” vs. “euphoria”), and asked to pick which picture corresponds to the person depicted in the previous story.

Previous research has conducted the same experiment in various cultures, and the results in different groups did not turn out the same. Accordingly, the scientist believes that people learn these emotions from experience with members of their own culture.

With this information in mind, how likely is it that, when presented with each emotion below, that the responses of the indigenous group would match those of participants in the US?

Please indicate your answer on a 1-7 scale (1= very unlikely; 7= very likely).

Thank you

**Appendix 2:**

**Emotions list**

| item | **Emotion** |
| --- | --- |
| 1 | Anger |
| 2 | Love |
| 3 | Contentment |
| 4 | Disgust |
| 5 | Excitement |
| 6 | Fear |
| 7 | Happiness |
| 8 | Joy |
| 9 | Pride |
| 10 | Sadness |
| 11 | Shame |
| 12 | Surprise |
| 13 | Contempt |
| 14 | Jealousy |
| 15 | Relief |
| 16 | Desire |
| 17 | Embarrassment |
| 18 | Pain |
| 19 | Awe |
| 20 | Boredom |
